# Effects of physiological aging factor on bone tissue engineering repair based on fetal BMSCs

**DOI:** 10.1186/s12967-018-1686-1

**Published:** 2018-11-23

**Authors:** Dingyu Wu, Zhenxing Wang, Zhiwei Zheng, Yingnan Geng, Zhanzhao Zhang, Qiannan Li, Quan Zhou, Yilin Cao, Zhi-Yong Zhang

**Affiliations:** 1 Department of Plastic and Reconstructive Surgery, Shanghai 9th People’s Hospital, Shanghai Key Laboratory of Tissue Engineering, School of Medicine, Shanghai Jiao Tong University, Shanghai, 200011 China; 2 Translational Research Centre of Regenerative Medicine and 3D Printing Technologies of Guangzhou Medical University, The Third Affiliated Hospital of Guangzhou Medical University, No. 63 Duobao Road, Liwan District, Guangzhou City, 510150 Guangdong Province China; 3 China Orthopedic Regenerative Medicine Group (CORMed), Hangzhou, 310058 China; 4 Department of Plastic Surgery, Union Hospital, Tongji Medical College, Huazhong University of Science and Technology, Wuhan, 430022 China; 5 Hunan Prevention and Treatment Institute for Occupational Diseases, Changsha, China

**Keywords:** Tissue engineered bone graft, Bone marrow stromal cells, Decalcified bone matrix, Aging factor, Bone tissue engineering

## Abstract

**Background:**

At present, many laboratories and hospitals all over the world are attempting and exploring the clinical transformation of this tissue engineered bone graft (TEBG) strategy. Many successful cases of bone tissue engineering (BTE) repair were based on young individuals. But there are little studies about the effectiveness of TEBG strategy in physiological aged individuals.

**Methods:**

In this research, we studied whether aging factor has influence on the skull repair effect of Fetal-TEBG, at the level of the large animal models. We used the fetal bone marrow stromal cells (Fetal-BMSCs) as the seed cells, combining the decalcified bone matrix (DBM) scaffolds, to repair the skull defects of the aged goats and the young goats. The repair effects on both aged goat and young goat were compared by Micro-CT and histology examination.

**Results:**

The skull defects of the young goats could be repaired better than that of the aged goats after 6 months by Fetal-TEBG; In the aged goats, although not completely repaired, the defects repaired by Fetal-TEBG was better than that repaired by the Control DBM scaffold.

**Conclusions:**

Aging factor has impact on the bone repair effect of Fetal-TEBG; and the BTE strategy is still efficacious even in the aged individuals. The improvement of the aged state may promote the repair effect of the BTE in the aged individuals. 
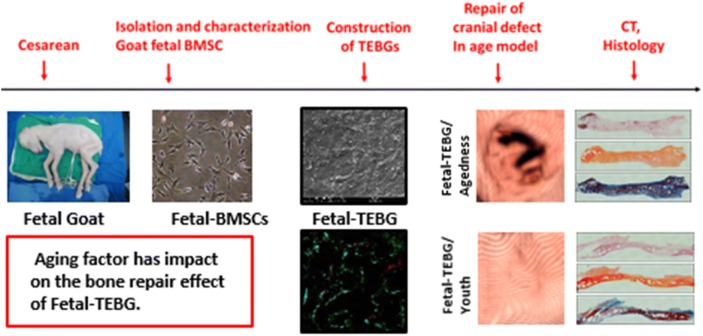

## Background

How to effectively repair large bone defects has always been a thorny clinical problem for orthopedics doctors. The tissue engineered bone repair strategy based on stromal cells (MSCs), is generally recognized as an effective solution to repair the large bone defects [1–9]. This strategy, combining MSCs with scaffolds, is used to construct tissue engineered bone graft (TEBG) in vitro for the repair of large bone defects. At present, many laboratories and hospitals all over the world are attempting and exploring the clinical transformation of this strategy.

Generally, many successful cases of BTE repair were based on young individuals [1–11]. However, aging population has become a worldwide phenomenon recently, and the treatment of bone defects in physiological aged individuals could also not be ignored [12–14]. Therefore, it is of great significance to study the BTE repair of bone defects in physiological aged individuals, and it could provide more experimental evidence for the later determination of clinical indicators.

But there are little studies about the effectiveness of TEBG strategy in physiological aged individuals. In this study, as the efficacy of autologous bone marrow stromal cells (Autologous-BMSCs) is significantly compromised in aged individuals, we used the Fetal-BMSCs as the seed cells [10, 11], combining the decalcified bone matrix (DBM) scaffolds, to repair the skull defects of the aged goats and the young goats. The repair effects on both aged goat and young goat were compared by Micro-CT and histology examination. Through this research, we studied whether aged factor has influence on the bone repair effect of Fetal-BMSCs, at the level of the large animal models; and we studied whether the BTE strategy is still efficacious in the aged individuals.

## Methods

### Isolation and identification of Fetal BMSCs

#### Isolation and culture of Fetal BMSCs

Institutional animal care committee approval was obtained for the study. Artificial fertilization was used to breed white goats (the goat was provided by the breeding base of Chongming Three Star Town, Shanghai, China, which were licensed by the Animal Experiment Committee of Medical School of Shanghai Jiaotong University). All experimental procedures were according to the institutional guidelines for the care and use of laboratory animals in Medical School of Shanghai Jiaotong University. Then the cesarean section was performed after 3 months pregnancy (the normal pregnancy of the goat is 5 months). Before operation, the goats were fasting for 2 days, and forbidden to water for 12 h. The goats was sedated by atropine half an hour before the operation. Tracheal intubation was performed after general anesthesia (intramuscular injection of xylazine). After skin preparation and iodophor disinfection, we cut the skin by electric knife, separated the subcutaneous fascia, opened the abdominal cavity, and took out the uterus. Finally, the fetal goats were taken out by cutting the uterus along base.

The limb bones of fetal goats were isolated in the sterile environment, and the ends of the limbs were cut off. Then the long bone marrow cavities were repeatedly aspirated with 5 mL syringes until the cavities appeared white. After that, the fresh bone marrow tissue was seeded onto 10 cm culture dishes with 10 mL of low glucose DMEM (Hyclone, Logan, UT, USA) supplemented with 10% FBS (Hyclone), and 1% penicillin and streptomycin (Thermo Fisher Scientific, Waltham, MA, USA). The culture dishes were incubated in a humidified environment (5% CO_2_, 37 °C) and the culture medium was changed every 3 days [15]. When the cells confluence reached 80%, 0.25% trypsin/1 mM EDTA (Thermo Fisher Scientific) was used to digest the MSCs (marrow stromal cells) for the passage until the third generation (P3).

#### Characterization of cells dynamics and identification of stem cells properties

##### Growth curve

When passaged to P3, the cells were seeded in 96-well plates at 1000 cells/well, then placed in a conventional incubator and incubated with cck-8 for 3 h on day 1, 3, 5, 7, 9, 11. With a micro-plate reader, the OD values of per hole and control hole were measured at 450 nm wavelength, and the difference value of the two was the final OD value. According to the final OD value, the cell growth curve was drawn.

##### Colony forming efficiency

The experimental procedure was the same as previously reported [10]. The Fetal BMSCs of P3 generation were evenly seeded into dishes at a cell density of 200 cells/dish. The colonies were formed after 21 days of culture.

##### Three induced differentiation ability

BMSCs (passage3) were passaged into 6-well plates at a cell density of 3 × 10^5^ cells/well. When the cells confluence reached 80%, the culture medium was replaced by induction medium [16]. For each plate, two wells were stained with Alizarin red S after 10 days’ osteogenic induction (10 mM β-glycerophosphate, 0.1 μM dexamethasone, and 50 μM ascorbic acid). Two wells were stained with Oil red O after 3 weeks’ lipid induction (5 μg/mL insulin, 200 μM indomethacin, 1 μM dexamethasone, and 0.5 mM 3-isobutyl-1-methylxanthine). Two wells were stained with Toluidine blue after 3 weeks of chondrogenic induction (DMEM, 10% fetal bovine serum, 0.1 μM dexamethasone, 0.17 mM ascorbic acid, 1 mM sodium pyruvate, 0.35 mM l-proline,1% insulin-transferrin sodium-selenite, 1.25 mg/mL bovine serum albumin, 5.33 μg/mL linoleic acid, and 0.01 μg/mL transforming growth factor-β).

### Construction of TEBG (tissue engineering bone graft)

#### Preparation of DBM

As previously described, the goat femur was decalcified and deproteinized to form decalcified bone matrix (DBM) [17]. The diameter of the goat skull defect was 20 mm, and according to that, the DBM scaffolds were fabricated by cutting the DBM into small discs (diameter 20 mm, thickness 2–3 mm).

#### Seeding cells

The DBM scaffolds were soaked in 75% alcohol for 5 h, and cleaned by PBS for 3 times. After dried up, the P3 generation BMSCs were then seeded on these DBM scaffolds at a cell concentration of 20 million/mL. After incubated in a humidified environment (5% CO_2_, 37 °C) for 4–5 h, the L-DMEM culture medium was carefully added along the dish wall until the scaffolds were covered by culture medium. On the second day, the culture medium was replaced by osteogenic induction medium, and the medium was changed every 3 days for 14 days.

#### Exam of cell viability by FDA/PI staining

After 7 days and 14 days of osteogenic induction, the cells of the TEBG were stained by fluorescein diacetate/propidium iodide (FDA/PI). The dyeing procedure was as previously described, FDA stained viable cells green and PI stained dead cells red [18].

#### Observation of TEBG before implantation by SEM

After 14 days of osteogenic induction, the TEBG was prefixed in 2.5% glutaraldehyde for 24 h at 4 °C. After being rinsed three-times in PBS, the specimens were immersed in 1% osmic acid for 2 h at 4 °C. And then rinsed three-times again in PBS. After drying, the specimens were sputter-coated with gold (BAL-TEC, Philips, Eindhoven, The Netherlands), and examined finally with a scanning electron microscope (PhilipsXL-30, The Netherlands) [10].

### BETG repair experiments in vivo

#### Repair of goats skull defects

##### Anaesthesia

The young group (2–3 years old normal adult female goat) and the aged group (10–12 years old, normal old female white goat) each had 6 goats, which were licensed by the Animal Experiment Committee of Medical School of Shanghai Jiaotong University. All animal work in this study was performed according to the institutional guidelines for care and use of laboratory animals in Medical School of Shanghai Jiaotong University. The preoperative fasting was 2 days, and water was forbidden for 12 h. Tracheal intubation was performed after general anesthesia (intramuscular injection of xylazine). Before the operation, the goat horn was sawed by a hand saw, leaving the roots, and bleeding was stopped by bone wax.

##### Defect model

The skin was cut along the midline of the craniofacial midline with electric knife. The subcutaneous fascia and muscle were separated and the bleeding was stopped by electrocoagulation. Then the top of the skull was exposed. The skull periosteum was stripped, and two skull defects of diameter 20 mm were drilled symmetrically at the top of the skull along midline. The skull bones that were drilled off were pried carefully, and residual skull fragments were removed by a bone nipper. The endocranium was retained.

##### Defect repair

After 14 days’ osteogenic induction, the TEBG was implanted into the left defect, while the Control DBM scaffold was implanted into the right defect as a control group. Then the muscle was sutured and the incision was closed after the TEBG and DBM were implanted in the defects. Three days after the operation, penicillin was injected for anti-infection.

#### CT scan and analysis

1.5 months, 3 months and 6 months after surgery, we anesthetized the goats and performed CT examination on the skull defects. After scanning, the skull was reconstructed with three-dimensional reconstruction software (VG studio Volume Graphics GmbH, Germany), and the bone volume (mm^3^) and bone volume/tissue volume ratio (BV/TV) were calculated. All these data were presented as mean ± SD.

#### Histology examination

6 months later, all the goats were over-anesthetized and euthanized in accordance with the animal experimental ethical standards of Medical School of Shanghai Jiaotong University. The specimens was separated and fixed by paraformaldehyde for 72 h. After decalcification of 0.6 M diluted hydrochloric acid, the specimens were sliced. As reported in previous studies [10, 19], the specimens were stained by H&E, VG and Masson respectively.

## Results

### Isolation and identification of fetal BMSCs

#### Cell morphology

**Fig. 1:**
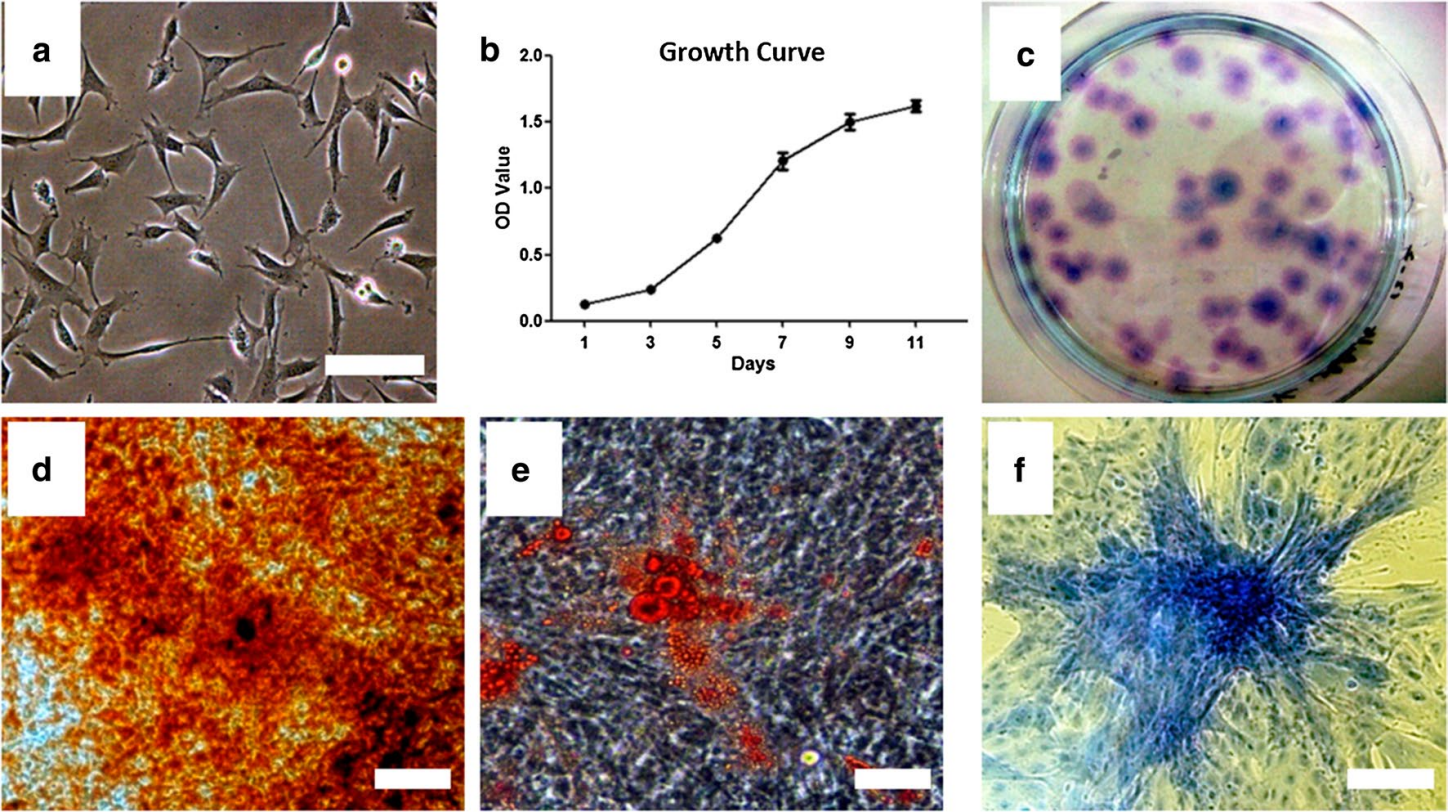
Isolate, culture and identify the fetal BMSCs of goats. **a** Morphology of fetal BMSCs were obtained from the goats; **b** the growth curve of fetal BMSCs (passage 3) on day 1, 3, 5, 7, 9, 11; **c** CFU of the fetal BMSCs after 21 days (stained by crystal violet); **d** Alizarin red S after 10 days’ osteogenic induction of BMSCs (passage 3); **e** Oil red O after 3 weeks’ lipid induction of BMSCs (passage 3); **f** Toluidine blue after 3 weeks’ chondrogenic induction of BMSCs (passage 3). Scale bar: **a** 25 μm; (**d**–**f**) 80 μm

The Fetal BMSC had “triangular” shape, with no obvious nuclei (aging characteristics), and its cytoplasm was homogeneous and dark (Fig. 1a).

#### Growth curve

In the proliferation test, the cells entered the logarithmic growth period from the 3rd day, and entered the platform stage between the 9th and 11th day. The growth curve appeared S shape, which was consistent with the growth characteristics of stem cells (Fig. 1b).

#### Colony formation units rate

Fetal BMSCs could form (49.2 + 4.3%) colonies, more than 30% (Fig. 1c).

#### Three induced differentiation ability

After the fetal BMSCs were induced by osteogenic differentiation, many red positive nodules were observed under alizarin red staining, with a larger number and larger area (Fig. 1d). This result suggested that the osteogenic capacity of fetal BMSCs was good. After the fetal BMSCs were induced by adipogenic differentiation, lipid droplets in the cells were dyed red by Oil red O staining (Fig. 1e). This result suggested that fetal BMSCs had a good lipid performance. After the fetal BMSCs were induced by chondrogenic differentiation, highly aggregated cell mass was dyed blue by toluidine blue staining (Fig. 1f). This result suggested that the fetal BMSCs had a good cartilage performance, containing rich glycosaminoglycan and collagen. From the above data, we can see the fetal BMSCs had the potential for multidirectional differentiation.

### Characterization of TEBG

**Fig. 2:**
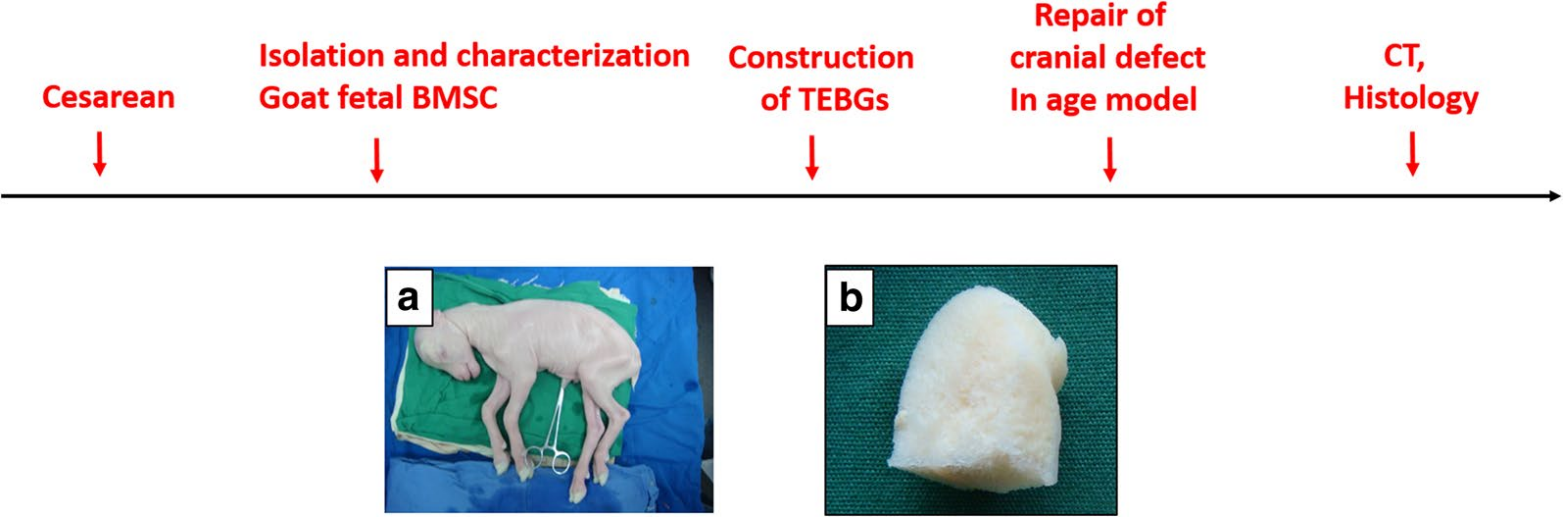
Schematic illustrations of the procedures for construction and the repair of cranial defect in age model. **a** Fetal goats; **b** DBM of goat femoral head obtained after degreasing and decalcification

**Fig. 3:**
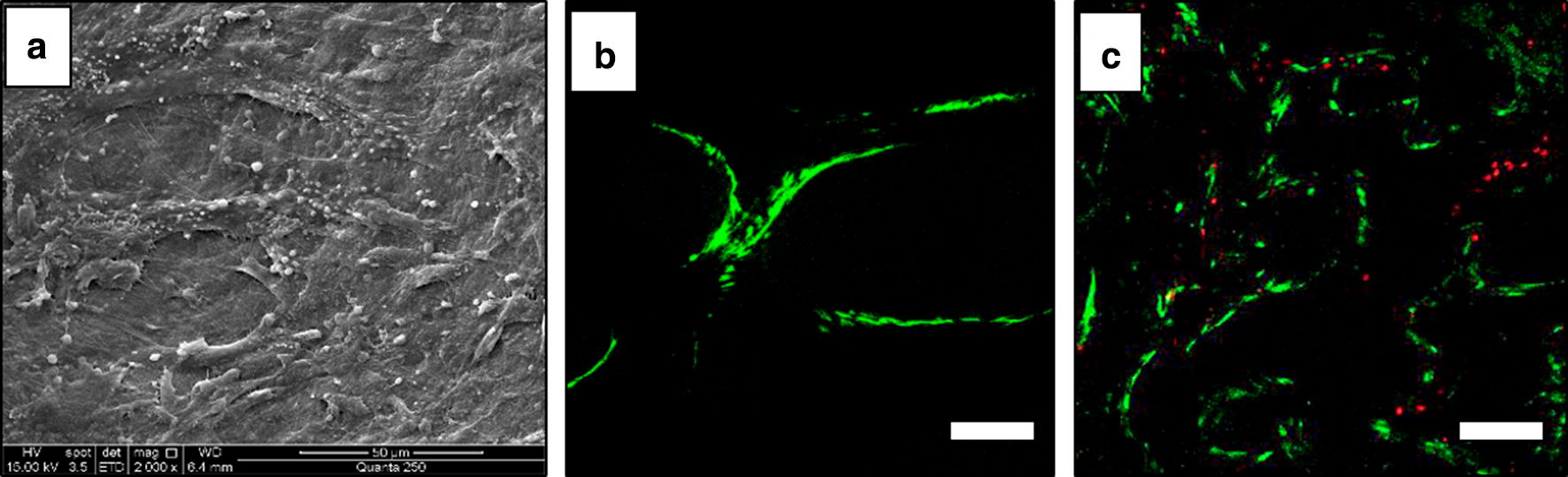
Characterization of Tissue engineering bone graft (TEBG). **a** TEBG observed in SEM after 14 days of culture; **b** FDP/PI staining of the TEBG after 7 days of culture; **c** FDP/PI staining of the TEBG after 14 days of culture. Scale bar: **a** 50 μm; **b**, **c** 80 μm

The DBM made from goat femoral head was soft and sponge like (Fig. 2b). Electron microscopy showed that all the cells on TEBG grew like paving stones, and the extracellular matrix was secreted vigorously, covering the scaffolds tightly and filling the pores (Fig. 3a). FDA/PI staining showed that the cells adhered to the edge of DBM aperture and grew well. In the field of vision, green cells (Live) were common and red cells (dead) were rare, which indicated that TEBG was well constructed (Fig. 3b, c).

### CT Imaging examination and analysis

**Fig. 4:**
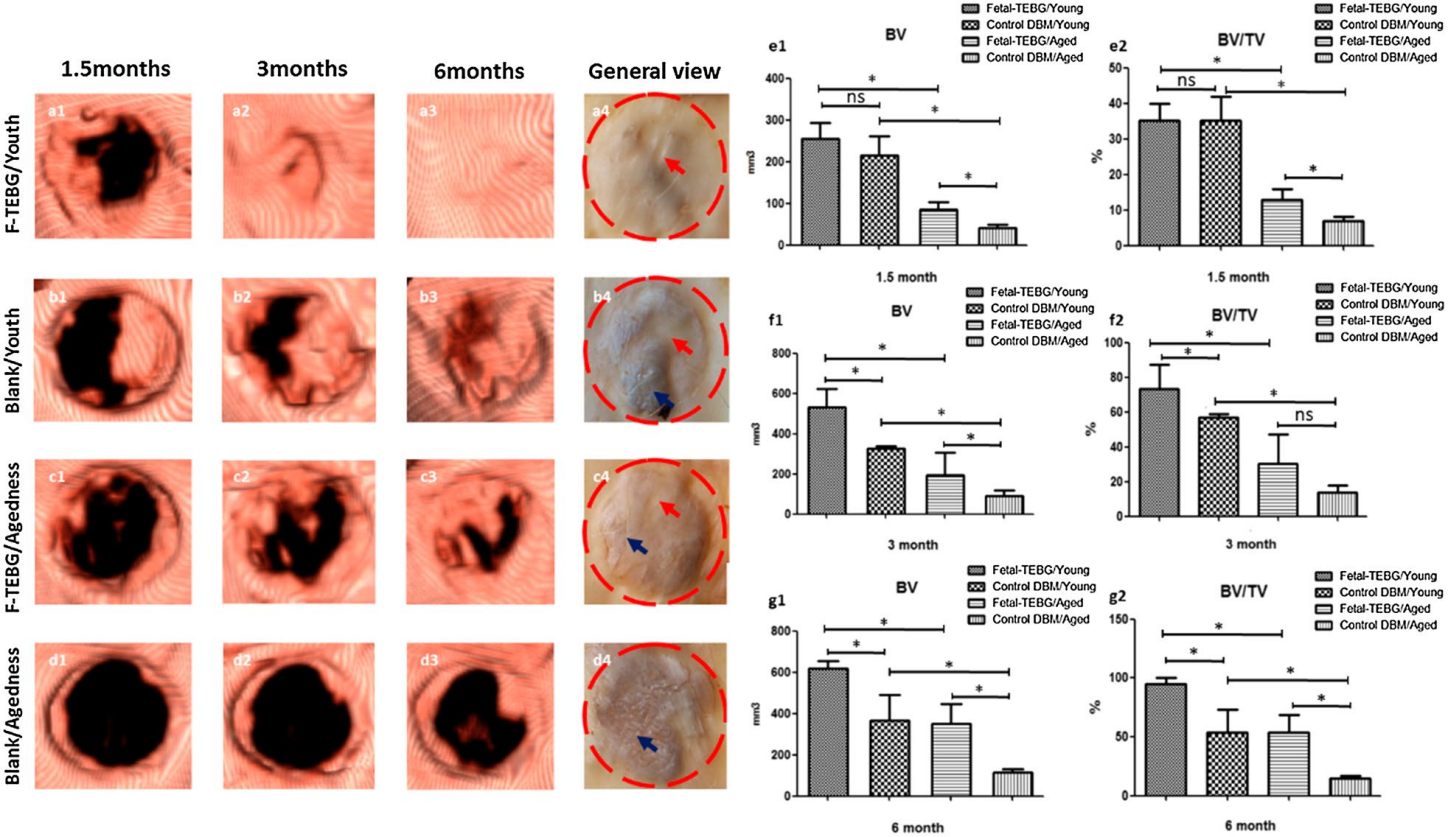
CT analysis and gross appearance of the repair of the goat skull defect. **a1**–**a3** 3D reconstruction of the CT of the cranial defect location repaired by Fetal-TEBG in young goats, 1.5 months, 3 months, 6 months. **a4** General view of the cranial defect location repaired by Fetal-TEBG in young goats. **b1**–**b3** 3D reconstruction of the CT of the cranial defect location repaired by Control DBM scaffold in young goats, 1.5 months, 3 months, 6 months. **b4** General view of the cranial defect location repaired Control DBM scaffold in young goats. **c1**–**c3** 3D reconstruction of the CT of the cranial defect location repaired by Fetal-TEBG in aged goats, 1.5 months, 3 months, 6 months. **c4** General view of the cranial defect location repaired Fetal-TEBG in aged goats. **d1**–**d3** 3D reconstruction of the CT of the cranial defect location repaired by Control DBM scaffold in aged goats, 1.5 months, 3 months, 6 months. **d4** General view of the cranial defect location repaired Control DBM scaffold in aged goats. **e1**, **e2** 1.5 months: the bone volume (BV) and bone volume/tissue volume ratio (BV/TV) in the skull defect area of Fetal-TEBG/Young group was more than that of Fetal-TEBG/Aged group (p < 0.05); In the Control DBM groups, the bone volume (BV) and bone volume/tissue volume ratio (BV/TV) of the young goats was also significantly higher than that of the aged goats (p < 0.05). **f1**, **f2** 3 months: the bone volume (BV) and bone volume/tissue volume ratio (BV/TV) in the skull defect area of Fetal-TEBG/Young group was more than that of Fetal-TEBG/Aged group (p < 0.05); In the Control DBM groups, the bone volume (BV) of the young goats was also significantly higher than that of the aged goats (p < 0.05). **g1**, **g2** 6 months: the bone volume (BV) and bone volume/tissue volume ratio (BV/TV) in the skull defect area of Fetal-TEBG/Young group was more than that of Fetal-TEBG/Aged group (p < 0.05); In the Control DBM groups, the bone volume (BV) and bone volume/tissue volume ratio (BV/TV) of the young goats was also significantly higher than that of the aged goats (p < 0.05)

After 1.5 months, 3 months, 6 months, the goats were scanned by CT and the data was analyzed and reconstructed by computer (Fig. 4). At the 1.5 months, the volume of bone formation in each group was as follows: Fetal-TEBG/Young: 255 ± 31 mm^3^; Control/Young: 216 ± 37 mm^3^; Fetal-TEBG/Aged: 85 ± 15 mm^3^; Control/Aged: 42 ± 6 mm^3^. At the 3rd months, the volume of bone formation in each group was as follows: Fetal-TEBG/Young: 530 ± 75 mm^3^; Control/Young: 326 ± 11 mm^3^; Fetal-TEBG/Aged: 195 ± 90 mm^3^; Control/Aged: 92 ± 21 mm^3^. At the 6th months, the volume of bone formation in each group was as follows: Fetal-TEBG/Young: 620 ± 27 mm^3^; Control/Young: 365 ± 103 mm^3^; Fetal-TEBG/Aged: 352 ± 77 mm^3^; Control/Aged: 115 ± 12 mm^3^. From the above data, we could see that the new bone volume in the Fetal-TEBG group was more than that of the control group, in both young goats and aged goats (p < 0.05). Most importantly, the bone volume in the skull defect area of Fetal-TEBG/Young group was more than that of Fetal-TEBG/Aged group (p < 0.05). In the control groups, the new bone formation of the young goats was also significantly higher than that of the aged goats (p < 0.05) (Fig. 4).

At the 1.5 months, the bone volume/tissue volume (BV/TV) in each group was as follows: Fetal-TEBG/Young: 35 + 4%; Control/Young: 35 + 5.6%; Fetal-TEBG/Aged: 13 + 2.3%; Control/Aged: 6.9 + 0.96%. At the 3rd months, the BV/TV in each group was as follows: Fetal-TEBG/Young: 73 + 11.5%; Control/Young: 57 + 1.6%; Fetal-TEBG/Aged: 30 + 13.8%; Control/Aged: 13.8 + 3.2%. At the 6th months, the BV/TV in each group was as follows: Fetal-TEBG/Young: 95.3 + 4%; Control/Young: 53.9 + 15.8%; Fetal-TEBG/Aged: 54 + 11.8%; Control/Aged: 15 + 1.8%. These data suggested that Fetal-TEBG’s repairing effect in both young goats and aged goats was significant. However, the skull defects of the young goats could be completely repaired after 6 months by Fetal-BMSCs, while the skull defects of the aged goats could not be completely repaired after 6 months (Fig. 4).

Under gross view, in the Fetal-TEBG/Young group, the white bone tissue (red arrow) covered almost all the repaired defects; in the Fetal-TEBG/Aged group, muscle and connective tissue (blue arrow) covered half part of the bone defects, and only half part of the bone defect was covered with white bone tissue (red arrow) (Fig. 4). And this was consist with the above data.

### Histology examination

**Fig. 5:**
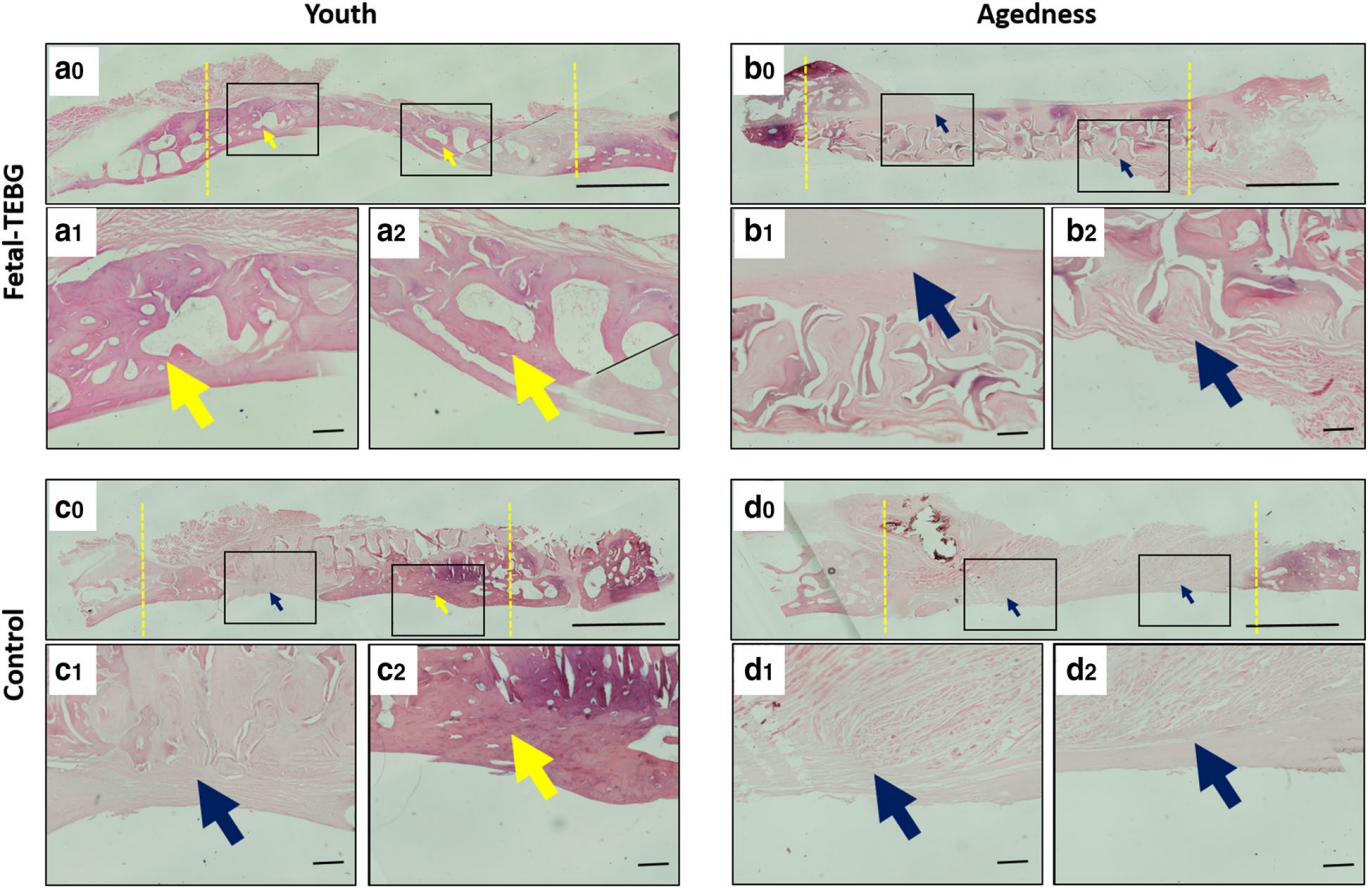
H&E (hematoxylin–eosin) staining of the four groups. **a0**–**a2** HE staining of the cranial defect location repaired by Fetal-TEBG in young goats. **b0**–**b2** HE staining of the cranial defect location repaired by Fetal-TEBG in aged goats. **c0**–**c2** HE staining of the cranial defect location repaired by Control DBM scaffold in young goats. **d0**–**d2** HE staining of the cranial defect location repaired by Control DBM scaffold in aged goats. (yellow arrow: bony tissue; blue arrow: fibrous connective tissue) Scale bar: (**a0**, **b0**, **c0**, **d0**) 500 μm; (**a1**, **a2**, **b1**, **b2**, **c1**, **c2**, **d1**, **d2**) 50 μm

HE staining showed that a dark red homogeneous bone tissue was formed in the bone defect (yellow arrow) in the Fetal-TEBG/Young group; In the Control/Young group, part of the bone defect area was occupied by dark red homogeneous stained bone tissue (yellow arrow), and part of the bone defect area was occupied by pink dyed fibrous connective tissue (blue arrow); In the Fetal-TEBG/Aged group, the defect was half covered with pink stained fibrous connective tissue (blue arrow), and the other half of the defect was covered with dark red homogeneous bone tissue; In the Control/Aged group, the defect was completely covered with pink stained fibrous connective tissue (blue arrow), and there was very little bone tissue (Fig. 5).

**Fig. 6:**
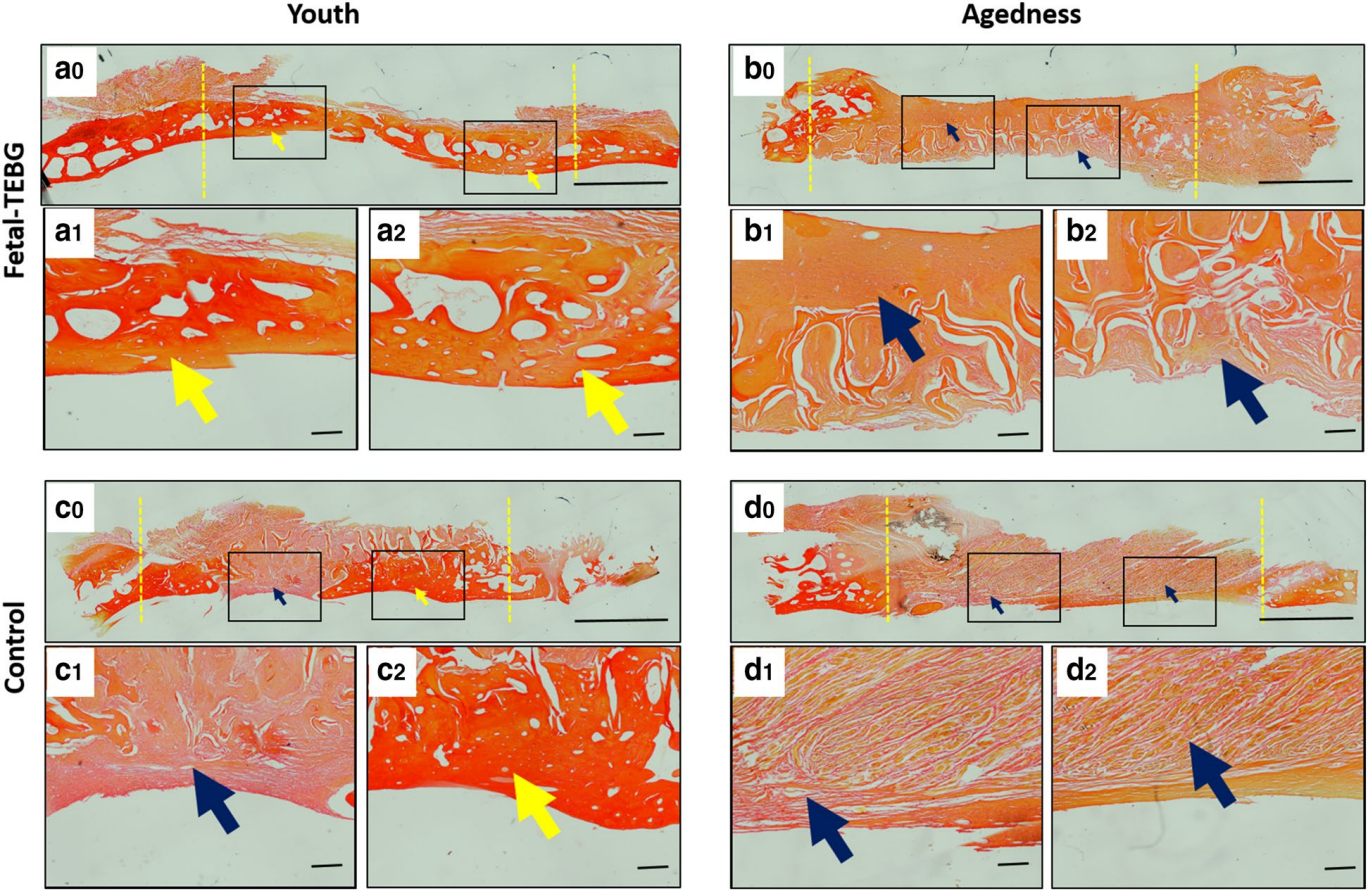
VG staining of the four groups. (**a0**–**a2**) VG staining of the cranial defect location repaired by Fetal-TEBG in young goats. (**b0**–**b2**) VG staining of the cranial defect location repaired by Fetal-TEBG in aged goats. (**c0**–**c2**) VG staining of the cranial defect location repaired by Control DBM scaffold in young goats. (**d0**–**d2**) VG staining of the cranial defect location repaired by Control DBM scaffold in aged goats. (yellow arrow: bony tissue; blue arrow: fibrous connective tissue) Scale bar: (**a0**, **b0**, **c0**, **d0**) 500 μm; (**a1**, **a2**, **b1**, **b2**, **c1**, **c2**, **d1**, **d2**) 50 μm

VG staining showed that there was a large amount of bright red homogeneous tissue (yellow arrow) in the Fetal-TEBG/Young group, suggesting new collagen tissue had formed; In the Control/Young group, part of the bone defect area was occupied by bright red homogeneous stained collagen tissue (yellow arrow), and part of the bone defect area was occupied by orange fibrous tissue and yellow connective tissue (blue arrow); In the Fetal-TEBG/Aged group, the defect was half covered with orange fibrous tissue and yellow connective tissue (blue arrow), and there was half part of the defect covered by dark red homogeneous stained collagen tissue (yellow arrow); In the Control/Aged group, the defect was completely covered with orange fibrous tissue and yellow connective tissue (blue arrow) (Fig. 6).

**Fig. 7:**
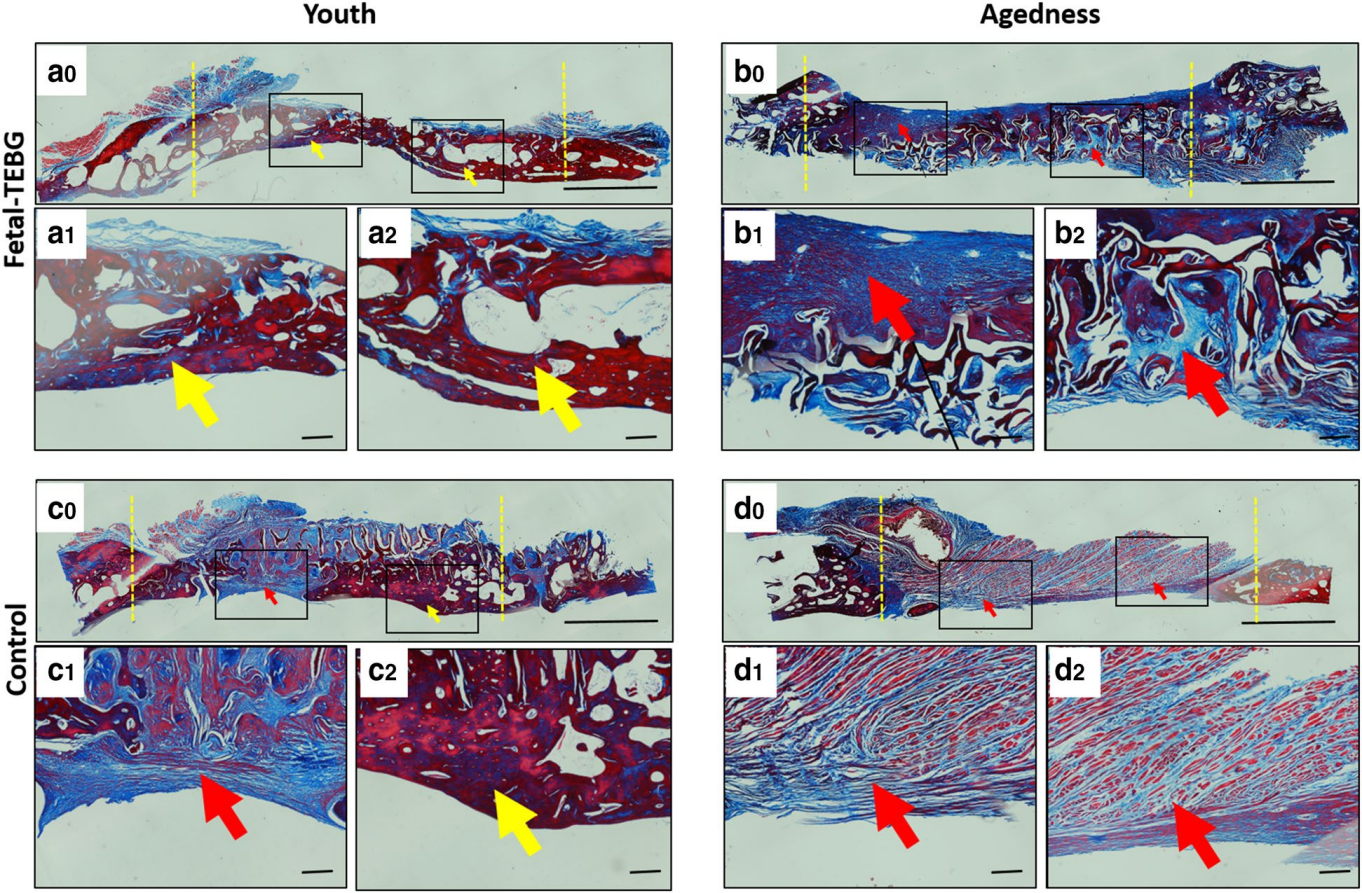
Masson staining of the four groups. (**a0**–**a2**) Masson staining of the cranial defect location repaired by Fetal-TEBG in young goats. (**b0**–**b2**) Masson staining of the cranial defect location repaired by Fetal-TEBG in aged goats. (**c0**–**c2**) Masson staining of the cranial defect location repaired by Control DBM scaffold in young goats. (**d0**–**d2**) Masson staining of the cranial defect location repaired by Control DBM scaffold in aged goats. (yellow arrow: bony tissue; red arrow: fibrous connective tissue) Scale bar: (**a0**, **b0**, **c0**, **d0**) 500 μm; (**a1**, **a2**, **b1**, **b2**, **c1**, **c2**, **d1**, **d2**) 50 μm

Masson staining showed that there was a large amount of dark red homogeneous bone tissue (yellow arrow) in the Fetal-TEBG/Young group, suggesting there was mature bone tissue formed in the area; In the Control/Young group, part of the bone defect area was occupied by dark red homogeneous stained bone tissue (yellow arrow), and part of the bone defect area was occupied by blue and light red fibrous connective tissue (red arrow); In the Fetal-TEBG/Aged group, the defect was half covered with blue and light red fibrous connective tissue (red arrow), and there was half part of the defect covered by dark red homogeneous stained bone tissue; In the Control/Aged group, the defect was completely covered with blue and light red fibrous connective tissue (red arrow) (Fig. 7).

## Discussion

The goat is a large mammal with a life span of 12–15 years. The number and structure of the bones in the limbs are similar to that of the human. It is a common preclinical research model [20]. In addition, previous experiments have proved that the goat is an animal model suitable for BTE research [21–23].

At present, the autologous bone marrow stromal cells (Autologous-BMSCs) are used the most widely in clinical transformation. However, in the aged individuals, the efficacy of the Autologous-BMSCs can be significantly compromised, making it least applicable in the elderly patients, for whom Autologous-BMSCs are mostly needed [24]. To solve the problem of the lower efficacy of Autologous-BMSCs effectively, we proposed the concept of “off the shelf” seed cells previously. Our previous study confirmed that the Fetal-BMSCs from abortion fetus are one kind of “off the shelf” stem cells [10]. It could repair bone defects effectively, and replace Autologous-BMSCs in BTE, for it had low immunogenicity [10]. Therefore we applied Fetal-BMSCs to this study directly.

In this study, the results of CT and histology showed that for the young individual, the Fetal-TEBG could repair the skull defect completely after 6 months, while for the aged individual, Fetal-TEBG could not repair the skull defect completely at the same time range. This indicates that the classical TEBG strategy has limited effect on the repair of the skull defect in physiological aged individuals. Therefore, it proved the aging factor has impact on the bone repair effect of Fetal-BMSCs based TEBG strategy. The results also suggest that in clinical transformation, the physiological state of aged individuals may not be conducive to the repair of bone defects. Meanwhile, in the aged groups, the new bone formation in the skull defect repaired by Fetal-BMSCs was also more obvious than that repaired by the aged goats. This indicates that although the physiological state is not conducive to the repair of the bone defects, the BTE strategy is still efficacious in the aged individuals.

It is generally believed that BTE repair contains two mechanisms: on the one hand, the implanted seed cells could effectively differentiate into new bone to repair defects; on the other hand, implanted cells can effectively mobilize recipient’s own stem cells to enter the defect site for bone repair [25]. In the aged individuals, the activity of endogenous stem cells are weaker, and the amount is smaller [25, 26]. Meanwhile, osteoporosis is common in aged individuals, and the microenvironment in the aged individual may not be conducive to the survival and osteogenic differentiation of exogenous seed cells [25–28]. Although we don’t know how the aging factor impact on the repair effect of Fetal-BMSCs, we could infer that, the artificial intervention, such as the effective improvement of the aged state and the osteoporosis microenvironment, might promote the repair effect of BTE effectively in the aged individuals. These are worthy of our further study.

## Conclusion

The skull defects of the young goats could be completely repaired after 6 months by Fetal-BMSCs, while the skull defects of the aged goats could not be completely repaired after 6 months. In the clinical transformation of BTE, we firstly proved that aging factor has impact on the bone repair effect of Fetal-BMSCs. How the aging factor impact on the repair effect, and how to improve the repair effect of the BTE strategy effectively in the aged individuals, are worthy of our further study.

## Abbreviations

TEBG: tissue engineered bone graft
BTE: bone tissue engineering
Fetal-BMSCs: fetal bone marrow stromal cells
DBM: decalcified bone matrix
MSCs: marrow stromal cells
Autologous-BMSCs: autologous bone marrow stromal cells
FDA/PI: fluorescein diacetate/propidium iodide
BV/TV: bone volume/tissue volume ratio
BV: bone volume
